# County-Level Variation in Per Capita Spending for Multiple Chronic Conditions Among Fee-for-Service Medicare Beneficiaries, United States, 2014

**DOI:** 10.5888/pcd13.160240

**Published:** 2016-12-01

**Authors:** Kevin A. Matthews, James Holt, Anne H. Gaglioti, Kim A. Lochner, Carla Shoff, Lisa C. McGuire, Kurt J. Greenlund

**Affiliations:** Author Affiliations: James Holt, Lisa C. McGuire, Kurt J. Greenlund, Centers for Disease Control and Prevention, Atlanta, Georgia; Anne H. Gaglioti, Morehouse School of Medicine, Atlanta, Georgia; Kim A. Lochner, Carla Shoff, Centers for Medicare and Medicaid Services, Baltimore, Maryland.

**Figure:**
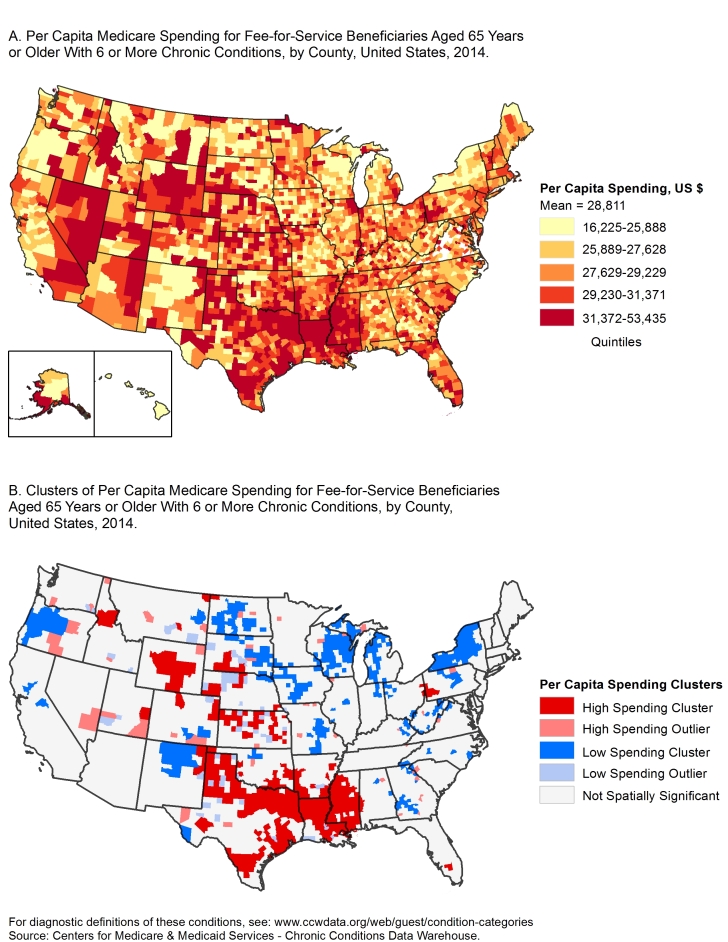
These maps show the geographic variation of standardized per capita Medicare spending, in US dollars, for all covered services among Medicare fee-for-service beneficiaries with 6 or more chronic conditions, United States, 2014. The maps highlight the need for targeted chronic disease prevention programs and policies in areas with the highest levels of Medicare spending.

## Background

The prevalence of Medicare beneficiaries aged 65 years or older with 6 or more concurrent chronic conditions (MCC6+) varies geographically (1). Preventing chronic disease costs less than treating it. Chronic diseases that are well managed progress slower than those that are untreated (2). Thus, understanding how Medicare spending is distributed across the United States among older adults with the highest burden of multiple chronic conditions can assist with targeting prevention and disease management efforts. The objective of this analysis was to describe the county-level variation in per capita Medicare spending among MCC6+ beneficiaries.

## Methods

We estimated per capita Medicare spending for MCC6+ beneficiaries aged 65 years or older by county using Centers for Medicare and Medicaid Services (CMS) enrollment and claims data for 100% of Medicare beneficiaries enrolled in the fee-for-service (FFS) program in 2014 (3). We excluded Medicare Advantage beneficiaries because claims data were not available; we also excluded beneficiaries enrolled in Medicare Part A only or Part B only, because their utilization was not comparable to those enrolled in both parts. The set of 19 chronic conditions examined — 1) hypertension, 2) hyperlipidemia, 3) arthritis, 4) ischemic heart disease, 5) diabetes, 6) chronic kidney disease, 7) heart failure, 8) depressive disorders, 9) chronic obstructive pulmonary disease 10) Alzheimer disease and related dementias, 11) atrial fibrillation, 12) cancer, 13) asthma, 14) stroke, 15) osteoporosis, 16) schizophrenia, 17) HIV/AIDS, 18) hepatitis (chronic viral B and C), and 19) autism — were those included in CMS’s public use files and that have been previously defined (4). A beneficiary was considered to have a chronic condition if there were claims with diagnosis codes indicating the beneficiary received a service or treatment of the condition. The number of claim years searched varied by condition as per previous methods (3).

Medicare spending included total Medicare payments for all Medicare-covered services in Parts A and B and was calculated per beneficiary (ie, per capita). Per capita Medicare spending in each county was derived by dividing total Medicare spending among the MCC6+ beneficiaries by the number of MCC6+ beneficiaries. We use standardized payments to account for the variation in Medicare payments for the same service in different areas. Standardized payments do not adjust for differences in health status among beneficiaries. More information on the standardization of Medicare payments is available (5).

CMS suppresses data with fewer than 11 beneficiaries per cell size. In this analysis, data on counties with fewer than 11 FFS beneficiaries with 6 or more conditions were suppressed (n = 38). For those counties, we imputed the number of beneficiaries by taking the difference between the number of beneficiaries in the state and the sum of beneficiaries in nonsuppressed counties, then distributing this difference equally across the suppressed counties in the state. The imputation process for Medicare spending for suppressed counties was similar, taking the difference between aggregate spending for the MCC6+ beneficiaries in the state and the sum of aggregate spending in nonsuppressed counties and distributing this difference equally across the suppressed counties in the state. To calculate a more stable per capita spending value, we included only counties that had at least 25 beneficiaries. For those counties with more than 11 and fewer than 25 beneficiaries, we calculated the distance from the center of each county to the centers of all other counties and used this distance to borrow cases from the nearest set of neighboring counties necessary to gain 25 beneficiaries. We classified county-level per capita Medicare spending into quintiles.

We used global and local Moran’s *I* to test for spatial autocorrelation (6) in per capita Medicare spending. Global methods indicate that clustering has occurred while local methods identify where the clusters were located. For both global and local measures, the Moran’s *I* statistic has values that range from −1.0 to 1.0. Generally, a Moran’s *I* with a positive value indicates that neighboring counties have similar values while dissimilar values are farther away, and a Moran’s *I* with a negative value indicates that neighboring counties have dissimilar values, like a checkerboard. For this study, the global Moran’s *I* tested whether per capita spending tended to cluster in the continental United States (6). The local Moran’s *I* identified 1) counties whose neighbors tended to have similar levels of spending, and 2) counties whose neighbors tended to have dissimilar levels of spending.

## Main Findings

In 2014, of the 28 million FFS beneficiaries aged 65 years or older, 4.3 million (15.4%) had 6 or more chronic conditions. Nationally, Medicare spending for this group totaled $126.1 billion, which accounted for 51% of Medicare FFS spending. Mean county spending was $28,811 per beneficiary (standard deviation, $3,879). Map A shows geographic variation in per capita Medicare spending.

The Moran’s *I* was 0.2509 (*P* < .001) indicating that counties in the continental United States with high spending levels tended to have neighboring counties with high spending, while counties with low spending levels tended to have neighboring counties with low spending. These geographic clusters are shown in Map B. Dark red areas are clusters in which counties with high spending are surrounded by counties with high spending. Dark blue areas are clusters where counties with low spending are surrounded by counties with low spending. Light red areas are counties with high spending but are surrounded by counties with low spending. Light blue areas are counties with low spending but are surrounded by counties with high spending. Northern Louisiana, western Alabama, northern Texas, and central Wyoming had the largest geographic concentrations of high per capita spending. Other areas with high spending were western Pennsylvania, Northeast Wisconsin, northwest Michigan, western Oregon and Upstate New York generally had low levels of spending.

## Action

These geographic differences in spending highlight the need to identify factors associated with the geographic pattern of spending for MCC6+ beneficiaries. These include risk factors for and costs associated with each of the 19 conditions and the most prevalent chronic conditions in a county. One approach to reduce spending is preventing chronic disease through preventive health care, public health policy, and public engagement in the noted geographic areas; targeted chronic disease management programs also could be beneficial in these areas, resulting in improved outcomes and reduced costs. Future work could include an exploration of beneficiaries’ care utilization patterns, such as the impact of chronic disease care delivered in primary care, hospital, and emergency settings on per-beneficiary costs.
